# Acute and Chronic Toxicity of Ketoprofen Active Pharmaceutical Ingredient and Commercial Formulations to the Freshwater Photosynthetic Species *Microcystis novacekii* and *Chlorella vulgaris*

**DOI:** 10.3390/ijerph23070829

**Published:** 2026-06-24

**Authors:** Gabriel Souza-Silva, Maria I. G. A. Silva, Anna C. B. Miranda, Mariângela Domingos Alcântara, Cléssius R. Souza, Micheline Rosa Silveira

**Affiliations:** Faculty of Pharmacy, Federal University of Minas Gerais, Belo Horizonte 31270-901, Brazil

**Keywords:** emerging contaminant, pharmaceutical waste, microalgae, cyanobacteria, anti-inflammatory, environmental health

## Abstract

**Highlights:**

**Public health relevance—How does this work relate to a public health issue?**

- The widespread environmental occurrence of the anti-inflammatory ketoprofen in surface waters and wastewater treatment effluents raises concerns about long-term exposure of aquatic ecosystems and, indirectly, human water supplies.
- The study focuses on freshwater photosynthetic microorganisms at the base of the aquatic food web, linking pharmaceutical contamination to potential disruptions of ecosystem services relevant to human well-being.

**Public health significance—Why is this work of significance to public health?**

- It demonstrates that ketoprofen and its commercial formulations exert ecotoxic effects on key primary producers at environmentally relevant exposure times, highlighting overlooked risks even for drugs considered low-toxic in classical acute tests.
- The inclusion of both the active ingredient and four different commercial formulations underscores the role of excipients and mixture effects, which may inform safer design and regulation of pharmaceutical products from a public health perspective.

**Public health implications—What are the key implications or messages for practitioners, policy makers and/or researchers in public health?**

- Regulatory and monitoring frameworks for pharmaceuticals in water should consider chronic exposure and sensitive freshwater species, not only acute toxicity and human pharmacology, to better capture ecosystem-linked public health risks.
- Results support the need for improved wastewater treatment technologies and eco-pharmacovigilance strategies, as well as further research on formulation-specific toxicity, to reduce environmental loads of ketoprofen and similar anti-inflammatory drugs.

**Abstract:**

Ketoprofen (KET) is a non-steroidal anti-inflammatory drug frequently detected in surface waters and effluents, with the potential to impact trophic base organisms. This study evaluated the toxicity of KET, in its active pharmaceutical ingredient (API) form and in four commercial formulations (KET-1, KET-2, KET-3, and KET-4), on two freshwater species: the cyanobacterium *Microcystis novacekii* and the microalga *Chlorella vulgaris*. Cell growth assays, performed under acute (4 days) and chronic (14 days) conditions, showed that the API KET was the most toxic compound, especially for *M. novacekii*, with a chronic EC50 of 1.35 mg/L. The commercial formulations presented distinct toxicity profiles, suggesting the influence of excipients and synergistic or antagonistic interactions. For *C. vulgaris*, low acute toxicity was observed, with increased chronic effects at high concentrations and possible hormetic response at low doses. Risk quotient (RQ) calculations, based on environmental concentrations of KET, indicated low risk in surface and drinking water, but high risk in untreated hospital and wastewater treatment plant effluents, especially for *M. novacekii*. The results show that the complete formulation, exposure time, and target species are critical factors in the ecotoxicological risk assessment of pharmaceuticals in freshwater environments.

## 1. Introduction

Nonsteroidal anti-inflammatory drugs (NSAIDs) constitute one of the most widely prescribed drug groups in the world [1,2,3]. Among these compounds, ketoprofen (KET) stands out as a drug with anti-inflammatory action mediated mainly by the inhibition of cyclooxygenase (COX) and lipoxygenase enzymes [4,5,6], being used by millions of patients per year [2].

Following oral administration, with a recommended daily dose of 200 mg per day, KET is rapidly absorbed [4,5,6] and conjugated with glucuronic acid via hepatic metabolism. Urinary elimination accounts for up to 90% of the administered dose, of which approximately 2% is excreted unchanged, and 60% is excreted as glucuronides [7].

The combination of several factors, such as high consumption [2], inefficiency of the sewage treatment system [8,9] and inadequate disposal of KET [10], makes this drug widely detected [4,11] in aquatic environments [4,9,12,13], soils [14] and river sediments [15] in concentrations ranging from ng/L to µg/L. Even at low concentrations, such compounds can cause significant adverse impacts on the ecosystem [4,8,11].

Thus, the increase in the consumption of pharmaceutical products represents a growing environmental challenge, especially due to the absence of specific regulations controlling their release and monitoring in the environment [4,9]. The main NSAIDs detected in the environment include KET, diclofenac, ibuprofen, and naproxen [16].

For decades, many in vivo pharmaceutical studies have pointed to the safety of consuming KET, with lethal doses (LD50) of approximately 100 mg/kg in rats [17]. Thus, it is assumed that consuming water containing environmental concentrations does not represent a direct and known risk to human health [18]. However, this anthropocentric view does not reflect reality, since KET residues, in environmentally relevant concentrations, can cause harm to other health systems, such as animal and environmental health, and consequently, to One Health [19]. These residues are associated with bacterial resistance, genetic and metabolic alterations in various aquatic organisms that are fundamental to life and environmental balance [4,8].

These environmental impacts caused by pharmaceutical waste, such as KET, fall directly within the scope of the One Health concept, which recognizes the interdependence between human, animal, and environmental health [20]. From an ecotoxicological point of view, NSAIDs contamination in aquatic ecosystems not only affects non-target organisms but can trigger a chain of adverse effects that reverberate throughout the ecological chain, ultimately impacting human health [4].

The presence of these compounds on the surface and groundwater, associated with bioaccumulation and biomagnification in human food [21,22], can compromise fundamental ecosystem services such as water quality and biodiversity maintenance. Waste from effluents can influence the formation of biofilms in drinking water distribution networks, reducing not only water quality but also its availability and consequently increasing the scarcity of a resource of paramount importance to life on the planet [23].

Therefore, among aquatic organisms of ecological importance, cyanobacteria and microalgae stand out for their wide distribution in aquatic environments [24]. These organisms play critical roles as producers in the trophic chain, acting in photosynthesis and nutrient cycling. However, the presence of these residues can cause effects on these organisms, favoring excessive growth, which can result in the production of toxins and harmful effects on the environment [25] or the inhibition of cell growth, reducing primary energy, that is, the food source of numerous species [26].

Despite the growing evidence regarding the occurrence and ecotoxicological effects of KET in aquatic environments, important knowledge gaps remain [4,11]. Most studies have focused on the active pharmaceutical ingredient (API), whereas aquatic organisms are more likely to be exposed to commercial pharmaceutical formulations containing excipients and other inactive ingredients that may alter the overall toxicity profile. Similar findings reported for pesticide formulations have demonstrated that co-formulants can substantially modify biological responses compared with the active ingredient alone, highlighting the importance of evaluating commercial products in ecotoxicological assessments [19,20].

Furthermore, information on the sensitivity of freshwater photosynthetic microorganisms to KET remains limited, particularly for *M. novacekii*, for which ecotoxicological data are scarce [24]. Comparative assessments involving cyanobacteria and green microalgae are also uncommon, limiting our understanding of species-specific susceptibility patterns. In addition, most available studies have focused on acute exposure scenarios, despite the continuous release of pharmaceuticals into aquatic ecosystems and the potential for chronic exposure at environmentally relevant concentrations. Consequently, the ecological risks associated with long-term exposure to KET and its commercial formulations remain poorly understood [4,8,9,11].

In this context, the present study aimed to evaluate the toxicological effects of KET on the freshwater organisms *M. novacekii* (cyanobacteria) and *C. vulgaris* (microalgae), belonging to taxonomic groups of ecological relevance in aquatic ecosystems. This research aims to contribute to the understanding of the environmental risks associated with the presence of NSAIDs in aquatic environments and to reinforce the importance of ecopharmacovigilance in mitigating environmental impacts.

## 2. Materials and Methods

### 2.1. Active Pharmaceutical Ingredient

The API KET, purity ≥ 98% (Sigma-Aldrich, St. Louis, MO, USA) used in this study was provided by the Center for Pharmaceutical Analytical Studies and Development (CEDAFAR) (Table 1).

### 2.2. Medicines

In addition to the API, four different commercial KET-based medications, registered by the pharmaceutical companies, were used: Sanofi Medley Farmacêutica Ltd.a, Suzano Brazil (KET-1), EMS S/A, São Bernardo do Campo, Brazil (KET-2), Momenta Farmacêutica Ltd.a, São Paulo, Brazil (KET-3), and Aché Laboratórios Farmacêuticos S/A, Guarulhos, Brazil (KET-4). For the tests, it was assumed that the actual concentration of KET is that stated by the manufacturer in the formulation, considering that the medications (active pharmaceutical ingredient + excipients) were tested in their entirety and approved by a rigorous pharmaceutical quality control system for commercial use. The composition of the medications is presented in Table 2.

Regarding the ratio between medication and active pharmaceutical ingredient, 10 units of the commercial medications were weighed, and the average weight of the tablets was obtained. For medication KET-1, the average weight was 330.2 ± 0.22 mg, with 100 mg of API, corresponding to approximately 30.3% of the tablet composition. For medication KET-2, the average weight of the medication capsules was 207.1 ± 0.12 mg, with 50 mg of API, corresponding to approximately 24.1% of the capsule composition. For medication KET-3, the average weight of the medication tablets was 829.7 ± 0.55 mg, with 150 mg of API, corresponding to approximately 18.1% of the tablet composition. Finally, for the drug KET-4, the average weight of the capsules was 570.0 ± 0.29 mg, with 320 mg of API, corresponding to approximately 56.1% of the capsule composition.

### 2.3. Test Substance Solubilization

To ensure osmotic balance between the biological model and the test substance, the different KET-based drugs were solubilized in ASM-1 culture medium at pH = 8.0 ± 0.2 [27] or BG-11 (supplemented with 0.02 M sodium nitrate) at pH = 7.5 ± 0.2 [28].

For solubilization, each medication was individually pulverized using a porcelain pestle and mortar. Then, all the material was transferred to a glass beaker and solubilized in each culture medium using a heating plate with magnetic stirring (IKA RT 15, Staufen, Germany), under controlled conditions of temperature (35 ± 1 °C), stirring (500 rpm) and time (10 min).

After reaching room temperature, the prepared solution (drug + culture medium) was filtered through a qualitative filter and the pH adjusted to pH = 8.0 ± 0.2 (ASM-1) or 7.5 ± 0.2 (BG-11) using a 0.1 M sodium hydroxide (NaOH) solution. The amount of medium used in the solubilization was considered the final concentration of the stock solution, i.e., 50 mg/L for each drug. From this stock solution, solutions with different concentrations were prepared and diluted in culture medium (Table 3).

### 2.4. Toxicity Assay

The assay to inhibit the growth of the cyanobacterium *M. novacekii* and the microalga *C. vulgaris* was conducted based on the recommendations of OECD guideline no. 201 (2011) [29], incorporating modifications to the exposure time, which was extended to 14 days in order to allow for the evaluation of chronic effects. Initially, 250 mL Erlenmeyer flasks were prepared containing the test concentrations, and algal cultures were inoculated to reach a starting density of 10^6^ cells/mL. The flasks were then incubated in triplicate at 22.0 ± 1.0 °C under a 12:12 h light/dark photoperiod for 96 h in the acute exposure assay and for 14 d in the chronic exposure assay.

ASM-1 and BG-11 media were used as negative controls, while a sodium chloride (NaCl) solution (6.0 g/L) served as the positive control. Exposure to 6.0 g/L NaCl resulted in 45 ± 5% and 25 ± 5% growth inhibition for *M. novacekii* and *C. vulgaris*, respectively, confirming the sensitivity of the test organisms.

During the 14-day chronic exposure period, cell density was maintained without dilution or alteration of the culture medium. Thus, the exposure was conducted in a static design. The exposure medium was not renewed throughout the experiment. To minimize evaporation during prolonged incubation, the containers were kept in BOD incubators and maintained under controlled conditions (temperature: 22.0 ± 1.0 °C; photoperiod: 12/12 h light/dark). No significant volume loss was detected. Cell growth was monitored only at the initial (0 days) and final (14 days) times by measuring optical density.

Cell growth was monitored by measuring optical density using a spectrophotometer (Spectroquant, Merck Millipore, Darmstadt, Germany) at 680 nm for *M. novacekii* and 695 nm for *C. vulgaris*. Cell density (cells/mL) was additionally determined by direct microscopic counting using a Neubauer chamber (Nikon Eclipse E200, Tokyo, Japan).

Y = 10^7^ × X − 310,552, (1)

Y = 10^5^ × X − 22,562, (2)

Based on the values of cellular optical densities, it was possible to evaluate the effects of different KET-based drugs regarding growth inhibition, growth stimulation, algicidal activity, and possible hormetic response.

In the cell growth inhibition assay, the effects on test organisms were evaluated by measuring cell biomass over time. Cell growth was monitored by optical density (680 or 695 nm) at 0, 4, and 14 days. From these data, the specific growth rate was calculated and compared with the negative control group, cells not exposed to the test substance. Growth inhibition was expressed as a percentage reduction relative to the control; that is, cell growth inhibition corresponded to values greater than 0 and less than or equal to 100.

For cell growth stimulation, the effects on test organisms were evaluated by measuring cell biomass over time (0, 4, and 14 days), as well as cell growth inhibition. This effect refers to the increased growth rate of cyanobacteria in response to KET exposure compared to the negative control, the unexposed group. Growth stimulation was expressed as a percentage reduction relative to the control; that is, cell growth stimulation was defined as values less than 0 (negative values) at all concentrations or partially at the highest concentrations.

In this study, the algicidal effect was the ability of KET to cause the death or direct degradation of the test organisms. Unlike growth inhibition, which can be reversible, the algicidal effect implies severe and irreversible cell damage. Cell growth was monitored by optical density (680 or 695 nm) at 0, 4, and 14 days. During the assay, this effect was detected by the decrease in cell density below the initial concentration. From these data, the specific growth rate was calculated and compared with the negative control group, as was cell inhibition. Thus, the algicidal effect was expressed as a percentage, i.e., values greater than 100.

Finally, the possible hormetic response, in turn, was considered a phenomenon characterized by a biphasic response to KET, in which, at low concentrations, the drug was able to stimulate cell growth or activity, while at higher concentrations, it caused cell inhibition. Based on these data, added to the requirement of the reported behavior, the possible hormetic response was calculated from the specific growth rate and compared with the negative control group, as with the other effects. Thus, the possible hormetic response was expressed as a percentage, i.e., values less than 0, meaning those that are negative at low concentrations.

### 2.5. Toxicity Classification on the Test Organism

Ecotoxicological assays involving cyanobacteria and microalgae commonly report the toxicity of a test substance in terms of cell growth inhibition, expressed as EC50 (half maximal effective concentration), following short-term exposure periods of 72 to 96 h. In contrast, chronic toxicity information is considerably more limited than acute data, and the lack of standardized protocols for long-term exposures leads to greater variability among studies, which complicates the assessment and classification of chronic environmental risk. In this study, for the classification of the toxicity of the test substance, both acute and chronic exposure, the adapted classification of the “ Hazardous “ standard was used to the aquatic environment” of Annex 2.28(B) of the Globally Harmonised System of Classification and Labelling of Chemicals [30] to determine the toxicity of the test substance on the *M. novacekii* or *C. vulgaris* (Table 4).

In this context, the GHS allows for a consistent and internationally recognized comparison of hazard levels between compounds, based primarily on acute toxicity data. However, it does not consider chronic toxicity or long-term ecological effects and, therefore, is used here as a complementary tool, not as a substitute for a comprehensive risk assessment. The GHS classification was applied to provide a standardized framework for comparing the intrinsic aquatic toxicity of the tested compounds based on EC50 values, allowing for a consistent classification of their hazard potential.

### 2.6. Environmental Risk Assessment—ERA

The environmental risk assessment was conducted using the ERA (Ecological Risk Assessment) framework [31]. In this study, the evaluation of ecological risk was based on Risk Quotients (RQs) (Equation (3)), which were calculated from the ratio between the measured environmental concentration (MEC), obtained from the Umweltbundesamt database [32], and the predicted no-effect concentration (PNEC) for KET. The PNEC values were derived from EC50 data generated in the present study, divided by an assessment factor (AF = 1000), as described in Equation (4).

RQs = MEC/PNEC,(3)

PNEC = EC50/AF,(4)

An evaluation factor was applied to ensure a conservative safety margin in estimating the environmental effects of anti-inflammatories based on experimental data. This approach considers the uncertainties associated with extrapolating acute to chronic effects, the variability between species, and the differences between laboratory conditions and the natural environment, as well as potential unforeseen adverse effects. Ecological risk was classified into four degrees according to the RQs value, as follows: RQs < 0.01, negligible risk; 0.01 ≤ RQs < 0.1, low risk; 0.1 ≤ RQs < 1, medium risk; and RQs ≥ 1, high risk.

### 2.7. Statistical Analyses

Statistical analyses were conducted using R software (version 4.5.1). Normality of the dataset was first assessed using the Shapiro–Wilk test. As the data did not satisfy normality assumptions, comparisons between each treatment and the negative control were performed using the Wilcoxon rank-sum test (Mann–Whitney U test). Differences were considered statistically significant at *p* < 0.05 (95% confidence level). Dose–response relationships were further evaluated using log-logistic, log-normal, and Weibull models. These models were implemented using the “drc” package in R, which was applied to identify the best-fitting model for the dataset [33].

Toxicity data (EC50) were statistically analyzed to assess significant differences between organisms, formulations, and exposures. First, we performed a two-way ANOVA (organism: *M. novacekii* vs. *C. vulgaris*; compound: KET-API, KET-1, KET-2, KET-3, KET-4) with experimental replication, using log-transformed EC50 (to normalize variances) and verifying assumptions of normality (Shapiro–Wilk) and homogeneity of variances (Levene).

The overall three-way ANOVA tested hypotheses of interaction (organism × compound × acute vs. chronic exposure), with an alpha of 0.05. In cases of significance (*p* < 0.05), we applied Tukey HSD post hoc tests for multiple comparisons, followed by Student’s *t*-tests for independent samples (*p* < 0.05, one-tailed, considering direction of effect) to compare EC50 between specific organisms (e.g., KET-API in *M. novacekii* vs. *C. vulgaris*, both for acute and chronic exposures).

## 3. Results

### 3.1. Toxicity of Ketoprofen in Cyanobacteria

**Figure 1:**
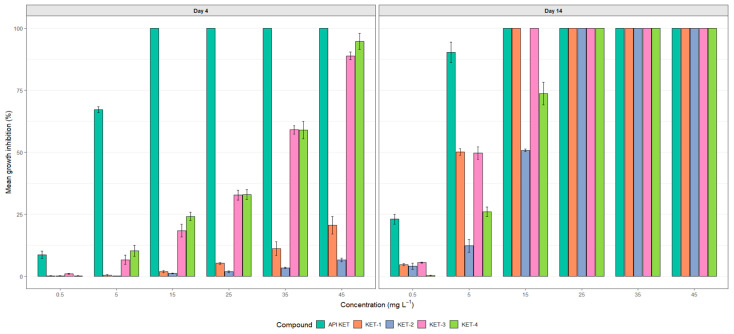
Concentration-dependent inhibition of cell growth in *Microcystis novacekii* exposed to ketoprofen (API and commercial formulations) under acute (4 days) and chronic (14 days) conditions.

Exposure of the cyanobacterium *M. novacekii* to different concentrations of KET resulted in significant inhibition of cell growth (Table 5, Figure 1). Regarding the effects observed from exposure to KET, inhibition of cell growth and an algicidal effect were observed after chronic exposure to the test substance in all drugs tested. However, none of the drugs tested showed a hormetic effect or stimulation of cell growth on this organism.

**Figure 2:**
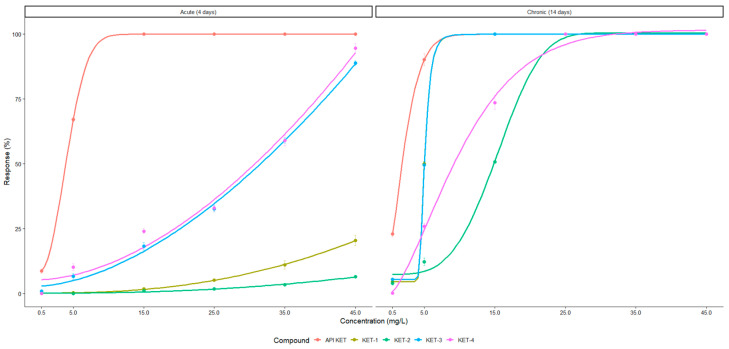
Sigmoidal dose–response curves describing growth inhibition of the cyanobacterium *Microcystis novacekii* exposed to ketoprofen (API KET) and commercial formulations (KET-1 to KET-4) under acute (4 days) and chronic (14 days) exposure. Curves were fitted using nonlinear regression models (log-logistic and Weibull functions) for each compound–time combination. Experimental points represent mean values of three replicates, and error bars indicate the standard error of the mean. Concentrations are expressed in mg/L. The *x*-axis shows nominal exposure concentrations, and the *y*-axis represents percentage growth inhibition relative to the control.

Among the solutions tested, the API KET exhibited the highest toxicity to the cyanobacterium *M. novacekii* (Figure 2). In acute exposure, concentrations above 0.5 mg/L caused significant cell inhibition (*p* < 0.05), while concentrations above 15.0 mg/L generated algicidal effects, eliminating approximately 90% of the cells; the EC50 was estimated at 3.22 ± 0.21 mg/L for concentrations between 0.5 and 15.0 mg/L. In chronic exposure, toxicity intensified, with EC50 reduced to 1.35 ± 0.15 mg/L and 5.0 mg/L, eliminating 25% of the cells. It was not possible to determine the NOEC (No Observed Effect Concentration), as the lowest concentration tested (0.5 mg/L) already inhibited cell growth, defining it as LOEC (Lowest Observed Effect Concentration).

KET-1 caused inhibition and algicidal effects, with toxicity dependent on time and concentration. In acute exposure, inhibition occurred above 0.5 mg/L, but was significant (*p* < 0.05) only above 15.0 mg/L; the highest concentration (45.0 mg/L) inhibited 20.4 ± 4.0% of the cells, preventing EC50 estimation, with a NOEC of 5.0 mg/L and an LOEC of 15.0 mg/L. In chronic exposure, significant inhibition (*p* < 0.05) occurred between 0.5 and 15.0 mg/L, with algicidal effects above 15.0 mg/L and an EC50 of 5.48 ± 1.41 mg/L (classified as “toxic”).

KET-2 induced inhibition and algicidal effects, also dependent on time and concentration. In acute exposure, inhibition occurred between 0.5 and 45.0 mg/L, significantly (*p* < 0.05) above 15.0 mg/L; the highest concentration (45.0 mg/L) inhibited only 6.9 ± 1.8% of cells, with no EC50 estimate, with a NOEC of 5.0 mg/L and a LOEC of 15.0 mg/L. In chronic exposure, significant inhibition (*p* < 0.05) occurred between 0.5 and 25.0 mg/L, with algicidal effects above 25.0 mg/L and an EC50 of 15.27 ± 1.07 mg/L (classified as “low toxicity”).

KET-3 caused significant cellular inhibition (*p* < 0.05) at all concentrations tested (0.5 to 45.0 mg/L) in acute exposure, with an EC50 of 32.44 ± 5.12 mg/L (“low toxicity”); the LOEC was 0.5 mg/L, with no NOEC observed. In chronic exposure, significant inhibition occurred between 0.5 and 15.0 mg/L, with algicidal effects above 15.0 mg/L and an LOEC of 5.85 ± 1.33 mg/L (“toxic”), with an EC50 of 0.5 mg/L and no NOEC.

KET-4 generated significant cellular inhibition (*p* < 0.05) at all concentrations (0.5 to 45.0 mg/L) in acute exposure, with an EC50 of 31.28 ± 3.44 mg/L (“low toxicity”); the LOEC was 0.5 mg/L, without any NOEC. In chronic exposure, significant inhibition occurred between 0.5 and 25.0 mg/L, with algicidal effects above 25.0 mg/L and an EC50 of 11.19 ± 0.82 mg/L (“low toxicity”), with an LOEC of 0.5 mg/L and no NOEC.

### 3.2. Toxicity of Ketoprofen in Microalgae

**Figure 3:**
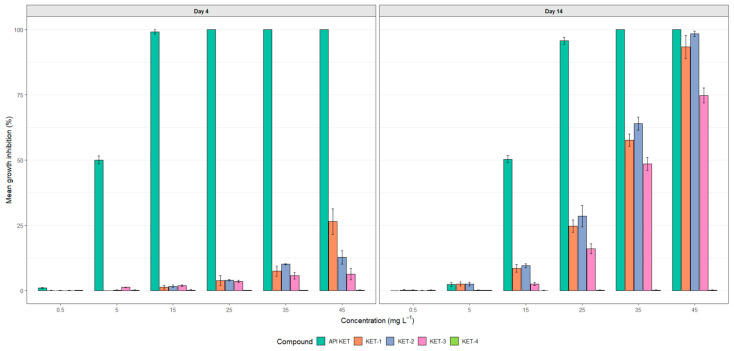
Concentration-dependent inhibition of cell growth in *Chlorella vulgaris* exposed to ketoprofen (API and commercial formulations) under acute (4 days) and chronic (14 days) conditions.

Exposure of the microalga *C. vulgaris* to different concentrations of KET resulted in significant inhibition of cell growth (Table 6, Figure 3). Regarding the effects observed from exposure to KET, only inhibition of cell growth was observed with commercial drugs, and inhibitory and algicidal effects were observed with exposure to the API. Furthermore, during chronic exposure, the API KET showed a possible hormetic effect, as it stimulated cell growth at low concentrations and inhibited growth at high concentrations. Among the commercial drugs evaluated, only KET-4 showed cell growth stimulation effects.

**Figure 4:**
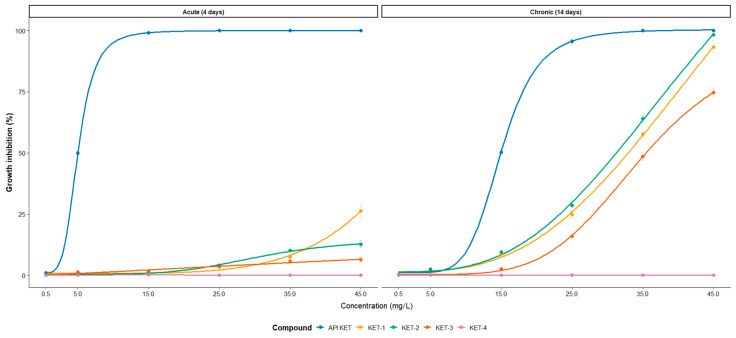
Sigmoidal dose–response curves describing growth inhibition of the microalgae *Chlorella vulgaris* exposed to ketoprofen (API KET) and commercial formulations (KET-1 to KET-4) under acute (4 days) and chronic (14 days) exposure. Curves were fitted using nonlinear regression models (log-logistic and Weibull functions) for each compound–time combination. Experimental points represent mean values of three replicates, and error bars indicate the standard error of the mean. Concentrations are expressed in mg/L. The *x*-axis shows nominal exposure concentrations, and the *y*-axis represents percentage growth inhibition relative to the control.

Although less intense compared to cyanobacteria, the API KET was the most toxic compound to the microalga *C. vulgaris* among those evaluated (Figure 4). In acute exposure, concentrations above 0.5 mg/L caused significant cell inhibition (*p* < 0.05), while concentrations above 15.0 mg/L generated algicidal effects, eliminating approximately 25% of the cells. The EC50 was estimated at 5.05 ± 0.66 mg/L (range 0.5 to 15.0 mg/L). In chronic exposure, toxicity decreased: concentrations below 5.0 mg/L stimulated cell growth, while concentrations between 5.0 and 25.0 mg/L inhibited growth, suggesting a possible hormetic response. The EC50 increased to 15.66 ± 1.34 mg/L, with algicidal effects only above 35.0 mg/L.

KET-1 caused time- and concentration-dependent cellular inhibition. In acute exposure, significant inhibition (*p* < 0.05) occurred above 15.0 mg/L, with 25.0 ± 5.0% inhibition at the highest concentration tested (45.0 mg/L), preventing the estimation of EC50; NOEC = 5.0 mg/L and LOEC = 15.0 mg/L. In chronic exposure, significant inhibition (*p* < 0.05) was observed between 5.0 and 45.0 mg/L, without algicidal effects, with an EC50 of 32.10 ± 3.22 mg/L (classified as “low toxic”).

KET-2 exhibited cellular inhibition similar to KET-1 (*p* > 0.05), also dependent on time and concentration. In acute exposure, significant inhibition (*p* < 0.05) occurred only above 25.0 mg/L, with 12.5 ± 2.5% inhibition at 45.0 mg/L, with no EC50 estimate, NOEC = 5.0 mg/L and LOEC = 15.0 mg/L. In chronic exposure, significant inhibition (*p* < 0.05) occurred between 5.0 and 45.0 mg/L, without algicidal effects, with an EC50 of 30.01 ± 2.45 mg/L (“low toxic”).

KET-3 caused significant cellular inhibition (*p* < 0.05) above 25.0 mg/L in acute exposure, with only 5.8 ± 2.2% inhibition at 45.0 mg/L, preventing the estimation of EC50; LOEC = 5.0 mg/L (NOEC = 0.5 mg/L). In chronic exposure, significant inhibition was observed between 15.0 and 45.0 mg/L, without algicidal effects, with an EC50 of 38.32 ± 2.00 mg/L (“low toxic”); NOEC = 5.0 mg/L and LOEC = 15.0 mg/L.

KET-4 did not induce cell inhibition at any exposure, showing only significant growth stimulation (*p* < 0.05) above 5.0 mg/L, dependent on time and concentration up to 25.0 mg/L (highest growth rate observed). The absence of toxicity prevented the estimation of EC50.

### 3.3. Comparative Analysis of Ketoprofen Sensitivity

The API KET demonstrated greater toxicity to the cyanobacterium *M. novacekii* than to the microalga *C. vulgaris* in both exposures. In the acute phase (4 days), the EC50 was 3.22 ± 0.21 mg/L for the cyanobacterium versus 5.05 ± 0.66 mg/L for the microalga, with chronic toxicity (14 days) intensifying more in the cyanobacterium (EC50 = 1.35 ± 0.15 mg/L, “toxic”) and decreasing in the microalga (EC50 = 15.66 ± 1.34 mg/L, “low toxic”). The cyanobacterium exhibited LOEC at 0.5 mg/L (no NOEC), while the microalga showed possible hormetic response at low chronic concentrations (<5.0 mg/L).

Among commercial drugs, cyanobacteria were consistently the most sensitive. KET-1 and KET-3 were “toxic” in chronic exposure to cyanobacteria (EC50 of 5.48 ± 1.41 and 5.85 ± 1.33 mg/L), but “low toxic” to microalgae (EC50 > 30 mg/L). KET-2 and KET-4 were “low toxic” to both, although KET-4 showed growth stimulation in microalgae (no EC50) and inhibition in cyanobacteria (chronic EC50 = 11.19 ± 0.82 mg/L). No compound caused possible hormetic response in cyanobacteria, unlike the API KET and KET-4 in microalgae. Algicidal effects occurred at lower concentrations in cyanobacteria.

### 3.4. Environmental Risk Assessment (ERA)

To assess the ecological risks of KET at environmental concentrations, we calculated risk quotients (RQ) for *M. novacekii* (more sensitive) and *C. vulgaris*. RQs were obtained by the MEC/PNEC ratio, using global maximum MECs (“worst case”). PNECs were derived from the lowest chronic EC50s of the API KET, adjusted by a safety factor of 1000: 1.35 µg/L for *M. novacekii* (EC50 = 1.35 mg/L) and 15.66 µg/L for *C. vulgaris* (EC50 = 15.66 mg/L) (Table 7).

RQs reveal a high risk for *M. novacekii* in most effluents and surface waters (RQ > 1.0), confirming critical vulnerability. For *C. vulgaris*, risks are high in effluents and superficial waters (RQ > 1.0), but low in potable/groundwater (RQ < 0.10). These values highlight hospital and wastewater treatment plant effluents as priority sources of contamination and require treatment for this pharmaceutical compound.

## 4. Discussion

The toxicological impact of pharmaceutical products is often attributed to APIs, but the contribution of excipients to environmental toxicity has been largely neglected, despite their essential presence in commercial formulations. Recent studies reinforce that certain excipients can be ecotoxicologically relevant, interfering with the bioavailability, biotransformation, or metabolism of drugs, in addition to acting as stressors in aquatic organisms [34].

In the present study, the different KET formulations (KET-1, KET-2, KET-3, and KET-4) demonstrated distinct toxicity profiles, even when the same active ingredient was used, similar to results observed by other authors [35]. This variability, coupled with the use of complete formulations (without quantitative decomposition of excipients), indicates that the observed toxicity cannot be attributed exclusively to the API, but also to interactions between the drug and the excipients, as well as to possible metabolic transformations during the assay [36]. The standardization of experimental conditions and triplicate repetition reinforce the consistency of the results, showing that the main variable was the manufacturer and, therefore, the composition and proportion of excipients.

Chronic exposure of *M. novacekii* to KET-1, for example, resulted in greater toxicity (EC50 = 5.48 mg/L, classified as “toxic”), while KET-2 and KET-4 remained in the “low toxic” category, and KET-3 showed similar behavior, being more toxic in chronic exposure (EC50 = 5.85 mg/L) than in acute exposure. These data suggest that the nature and proportion of excipients, such as sodium lauryl sulfate and povidone, may modulate the toxicity of KET, either through synergistic (KET-1), antagonistic (KET-2), or attenuating (KET-4) effects [34].

Data from the scientific literature regarding drugs of the same class but different formulations corroborate this pattern, showing that excipients can act as enhancers or mitigators of environmental toxicity. Studies with the same pharmaceutical active ingredient and different excipients show variations of up to an order of magnitude in EC50 values, reinforcing the need to evaluate complete formulations instead of just the isolated active ingredient [36,37].

Among the organisms tested, *M. novacekii* proved to be more sensitive to KET than *C. vulgaris*, both in terms of EC50 and the magnitude of the observed effects. For the API KET, the chronic EC50s were 1.35 mg/L for *M. novacekii* and 15.66 mg/L for *C. vulgaris*, which highlights the greater vulnerability of cyanobacteria, especially in chronic or high-concentration scenarios. This greater sensitivity reflects the lower tolerance capacity of many cyanobacteria to oxidative stress and chemical stressors, while *C. vulgaris* exhibits a more robust physiology to some xenobiotics [8,38].

Nevertheless, the microalga cannot be considered “insensitive”. In chronic exposure, the API KET exhibited inhibition of cell growth at higher concentrations, with algicidal effects above 25–35 mg/L, and possible hormetic response at low concentrations, with stimulation of cell growth between 0.5 and 5.0 mg/L. This possible hormetic response behavior, described in other studies with anti-inflammatory agents in *C. vulgaris* [39], indicates that the toxicological-ecological response is non-linear, and may, within certain ranges, favor the growth of tolerant microalgae even in contaminated environments.

Among the commercial medications, KET-1, KET-2, KET-3, and KET-4 showed chronic EC50 values greater than 30 mg/L for *C. vulgaris*, and only KET-4 showed growth stimulation, without significant inhibition. In contrast, these same formulations were able to generate algicidal effects on *M. novacekii*, even at 15 mg/L, which highlights the importance of considering model and trophic species (cyanobacteria vs. green algae) in risk assessment, since the loss of cyanobacteria can remodel entire trophic chains in freshwater ecosystems [40].

The study results underscore that the toxicity of KET is time- and concentration-dependent, with significant effects often only becoming evident after chronic exposure. For KET-1 and KET-2, it was not possible to estimate EC50 in acute exposure, as the inhibitory effect remained below the detection limit at the tested concentrations, while in chronic exposure, the average EC50 values ranged between 30 and 32 mg/L for *C. vulgaris* and 5–15 mg/L for *M. novacekii*. This dynamic reinforces the idea that compounds with low acute toxicity can present significant risks in prolonged exposures, especially in smaller organisms such as cyanobacteria and microalgae [34,41].

Furthermore, the persistence of KET in aquatic environments, coupled with its ability to undergo photodegradation and hydrolysis [19], can generate byproducts with greater stability and toxic potential [42]. Photodegradation of KET under sunlight produces compounds such as 3-hydroxyethylbenzophenone, 3-hydroperoxyethylbenzophenone, 3-acetylbenzophenone, and 3-ethylbenzophenone, which may exhibit distinct or even greater toxicity than the original molecule [43]. In the context of this study, conducted in a 12/12 h light/dark cycle, it is plausible that the presence of light radiation contributed to the formation of active metabolites, amplifying the observed inhibitory effects, especially in chronic exposures.

Environmental risk assessment using RQ, based on actual environmental concentrations of KET in drinking water, surface water, groundwater, hospital wastewater, and wastewater treatment plant effluent, showed that the greatest risks are associated with untreated effluents, with RQ values much higher than 1 for both *M. novacekii* and *C. vulgaris*. Importantly, surface water was also classified as presenting a high ecological risk (RQ > 1) for both organisms, indicating that environmentally relevant concentrations of KET may adversely affect primary producers in natural freshwater ecosystems. Because surface waters constitute the primary receiving environments for pharmaceutical contamination, these findings highlight the ecological relevance of KET exposure beyond wastewater sources [19,42]. Even so, the cyanobacterium remains the most sensitive organism, with high RQ values even at relatively low concentrations, highlighting the risk of cyanobacterial suppression in freshwater systems [44].

The greater sensitivity of *M. novacekii* compared to *C. vulgaris* is also in line with comparative studies with other cyanobacteria and green algae, which show greater vulnerability of cyanobacteria to the presence of anti—inflammatory drugs, especially in non-saline freshwater systems [45]. Because the solubility, ionization state, bioavailability, and uptake of ionizable pharmaceuticals can vary with salinity and other water chemistry parameters, toxicity data generated in marine or estuarine environments may not be directly representative of freshwater systems, where exposure and biological effects can differ substantially [46]. The identification of high ecological risk in surface waters is particularly concerning because these environments support diverse microbial communities that play fundamental roles in primary production, nutrient cycling, and ecosystem functioning [4,11]. The presence of cyanobacteria in these systems, often responsible for potentially toxic blooms, reinforces the relevance of monitoring not only the concentration of drugs but also the composition and dynamics of microbial communities [47].

*C. vulgaris* is widely used as a test organism in ecotoxicity assays because it is a photosynthetic green algae species that is easy to cultivate, has high growth reproducibility, and moderate sensitivity to various chemical stressors [48]. Previous studies [49,50,51,52] with anti—inflammatory drugs in *C. vulgaris* show that even at environmentally plausible concentrations, it is possible to observe inhibition or stimulation of cell growth, which is consistent with the results obtained here with KET and its formulations.

The observation of growth stimulation in *C. vulgaris* at KET-4, for example, may have complex ecological implications: if the microalga becomes more competitive in contaminated environments, this may alter the composition of phytoplankton communities, favoring certain photosynthetic groups over others, such as more sensitive cyanobacteria [53]. In the long term, these changes in the primary productivity base may impact the structure of trophic chains, as well as the capacity to regulate eutrophic balance, especially in systems already under organic load pressure.

### 4.1. Species-Specific Sensitivity and Physiological Mechanisms

The differential sensitivity observed between *M. novacekii* and *C. vulgaris* in response to KET constitutes one of the most ecologically informative findings of this study and warrants mechanistic interpretation beyond a mere descriptive comparison of EC50 values. *M. novacekii* exhibited consistently greater sensitivity to KET than *C. vulgaris* across both exposure periods. The chronic EC50 for the API reached 1.35 mg/L for the cyanobacterium compared to 15.66 mg/L for the microalga, a difference of more than one order of magnitude, and this divergence was statistically confirmed by the three-way ANOVA and subsequent post hoc tests.

Two-way ANOVA analysis (organism × compound × exposure) revealed significant differences in toxicity (F = 28.45; *p* < 0.001). Post hoc tests (Student’s *t*-test) confirmed significantly lower EC50 values for *M. novacekii* versus *C. vulgaris* in the API KET (acute: t = 3.12, *p* = 0.01; chronic: t = 12.34, *p* < 0.001) and chronic KET-1/3 (*p* < 0.05). There were no differences for KET-2/4 (*p* > 0.05).

This pattern likely reflects fundamental differences in cell wall architecture, membrane composition, and antioxidant capacity between the two phylogenetic groups. Cyanobacteria lack the true membrane-bound nucleus and the compartmentalized organelle structure present in eukaryotic algae; their photosynthetic machinery is embedded directly in the cytoplasm through thylakoid membranes, without the protective envelope of the chloroplast [54,55,56].

Consequently, membrane-active compounds and reactive oxygen species (ROS) generated during drug metabolism have more direct access to the photosynthetic apparatus in cyanobacteria [38,54,55]. KET, as a lipophilic weak acid (log K_OW_ = 3.1; pKa = 4.4) [57], is capable of crossing lipid membranes and, particularly at the test medium pH of 8.0 used for *M. novacekii*, exists predominantly in its ionized form. The balance between ionized and neutral species governs cellular uptake; however, even in its ionized form, KET can be incorporated into cells via passive diffusion of the unionized fraction or through the disruption of membrane integrity, a process potentially more damaging in prokaryotic membranes, which lack the sterol-based structural reinforcement found in eukaryotic cells [46].

Additionally, the antioxidant defense systems in cyanobacteria are comparatively less diversified than those in green microalgae. *C. vulgaris* possesses a well-characterized battery of enzymatic antioxidants, including superoxide dismutase, catalase, and ascorbate peroxidase, as well as non-enzymatic mechanisms such as carotenoids and tocopherols, which collectively confer greater resilience to oxidative insults induced by xenobiotics [38,53].

The hormetic response observed exclusively in *C. vulgaris* during chronic exposure to the API KET (growth stimulation at concentrations below 5.0 mg/L, followed by inhibition at higher doses) is consistent with this interpretation: at sub-inhibitory concentrations, upregulation of antioxidant defenses and adaptive metabolic responses may transiently stimulate cell division, a phenomenon previously reported for anti-inflammatory drugs in green algae [39]. The complete absence of possible hormetic response in *M. novacekii* reinforces the hypothesis that cyanobacteria lack the physiological plasticity to mount a compensatory adaptive response at sublethal KET concentrations [58].

From a mechanistic standpoint, KET inhibits cyclooxygenase (COX) and lipoxygenase enzymes in mammals; while orthologous enzymes in the classical sense are absent in cyanobacteria and microalgae, fatty acid oxygenase pathways with functional similarity to COX have been described in cyanobacteria [8,25]. Disruption of such pathways could impair membrane lipid homeostasis and signaling, contributing to the greater susceptibility of *M. novacekii* [38,54,55].

Furthermore, cyanobacteria are known to be particularly sensitive to compounds that interfere with photosystem II (PSII) activity, since their light-harvesting phycobilisome complexes are exposed on the cytoplasmic surface of the thylakoid membrane [38,54,55]. Although the precise mode of action of KET on PSII in cyanobacteria remains to be directly demonstrated, the algicidal effects observed at concentrations as low as 5 mg/L in chronic exposure, manifested as cell density falling below the initial inoculum, are indicative of severe photosynthetic impairment rather than mere growth retardation.

Regarding the ecological relevance of the EC50 values obtained, the chronic EC50 of 1.35 mg/L for *M. novacekii* is notably low relative to environmental concentrations reported for KET in untreated hospital effluents and WWTP, where concentrations can reach hundreds of micrograms per liter and, in exceptional cases, values in the mg/L range [32].

The calculated Risk Quotients (RQ > 1.0 for *M. novacekii* in surface water worst-case scenarios and in effluents) confirm that ecologically meaningful risks exist under real exposure conditions, particularly in freshwater systems receiving inadequately treated pharmaceutical waste [8,9]. The suppression of cyanobacteria in these environments carries consequences that extend well beyond the populations directly exposed: cyanobacteria are key contributors to nitrogen fixation in many freshwater ecosystems, and their loss may cascade into reduced nitrogen availability for primary production, altered phytoplankton community structure, and shifts in zooplankton food quality [40,44,47].

In productive, nutrient-rich systems where cyanobacterial blooms are already a concern, the selective pressure exerted by KET on *M. novacekii*-type organisms could paradoxically favor bloom-forming taxa with greater intrinsic resistance, or alternatively suppress nitrogen-fixing cyanobacteria, with complex and unpredictable outcomes for ecosystem functioning [8].

It is important to note that the differences in toxicity profiles between commercial formulations (KET-1 through KET-4) and the API, while real and statistically supported, must be interpreted with appropriate caution given the experimental design of this study. The formulations were tested as intact preparations without individual quantification of excipients, and the actual KET concentration in each test solution was assumed based on manufacturer declarations rather than confirmed analytically.

However, KET is generally considered a chemically stable compound under standard laboratory conditions, showing low susceptibility to hydrolysis in neutral to slightly alkaline aqueous media and limited degradation in the absence of strong photolytic or oxidative stress. Therefore, substantial losses of the active ingredient during short-term dissolution and exposure under controlled laboratory conditions are not expected. Nevertheless, potential variability associated with dissolution efficiency, filtration procedures, and formulation-specific characteristics cannot be completely excluded [59,60,61].

As a result, attributing the observed inter-formulation differences in EC50 to specific excipient-API interactions, whether synergistic or antagonistic, is not possible within the current dataset. What can be stated with confidence is that the observed variability in biological response across formulations is real and reproducible under the standardized experimental conditions applied, and that it justifies further investigation through designs that include individual excipient testing and analytical verification of nominal concentrations. The present findings therefore provide a basis for hypothesis generation rather than mechanistic attribution concerning formulation-specific effects.

### 4.2. Study Limitations and Future Perspectives

This work can be recognized as useful concerning the data collected on acute and chronic toxicities of API KET and commercial formulations to freshwater photosynthetic microorganisms. Nevertheless, a few limitations could be stated concerning the interpretation of the findings obtained from this study. In the first place, even though certain toxicological differences were established between the tested API and its commercial formulations, it is worth noting that the concentration of excipients in each formulation was not estimated, which does not allow us to specify which specific excipients caused certain biological responses.

Additionally, the chronic toxicity assays were conducted under static conditions for 14 days without medium renewal. Although no measurable volume loss was observed during the experimental period, prolonged incubation may have resulted in gradual nutrient depletion and the accumulation of metabolic byproducts released by the microalgae. These factors may have influenced growth dynamics independently of KET exposure and could have contributed to the growth responses observed, particularly at lower concentrations. Therefore, the chronic toxicity estimates should be interpreted considering the potential influence of these culture conditions.

Consequently, the involvement of excipients in synergy or antagonism of the studied pharmaceuticals remains an assumption. Moreover, actual concentrations of KET in the test solutions prepared according to the manufacturers’ instructions were not confirmed through analytical methods. Moreover, the generation of the transformation products was not investigated in order to estimate their contribution to the biological effects observed in the study. Future studies should aim at investigating commercial formulations of drugs from the pharmacological and toxicological perspectives and estimate the contribution of excipients to observed biological effects and identify transformation products, and evaluate toxicity under semi-static or flow-through exposure systems to minimize potential effects associated with nutrient depletion and metabolite accumulation.

## 5. Conclusions

The results of this study show that KET, in its different formulations, exerts toxic effects on the cyanobacterium *M. novacekii*, with greater sensitivity in chronic exposure conditions and variations in toxicity between formulations. However, these differences should be interpreted with caution, as the potential contribution of excipients, interactions between formulation components, and environmental degradation products cannot be confirmed in the absence of direct quantification or analytical characterization. In addition, although *C. vulgaris* showed lower sensitivity compared to the cyanobacterium, chronic exposure to KET and its commercial formulations resulted in growth inhibition at higher concentrations, as well as hormetic and growth stimulation responses at lower concentrations, demonstrating that the ecological effects of this pharmaceutical are species-dependent and may alter phytoplankton community dynamics. The persistence of the drug in aquatic compartments and its low removal in treatment plants reinforce the need for ecopharmacovigilance strategies and more comprehensive trials, considering not only the active ingredient but also the complete formulation and key phytoplankton organisms, to protect the structure, biodiversity, and functioning of freshwater ecosystems.

## Figures and Tables

**Table 1:** Physicochemical properties of ketoprofen and ketoprofen lysineate.

Information	Ketoprofen	Ketoprofen Lysineate
Chemical structure:	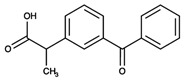	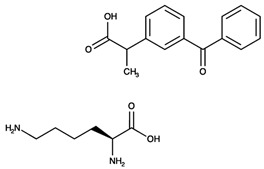
CAS:	22071-15-4	57469-78-0
Molecular weight:	254.28 g/mol	400.5 g/mol
Chemical formula:	C16H14O3	C22H28N2O5
pKa:	4.4	3.9
Log KOW:	3.1	3.3
Solubility in water:	51 mg/L	250 mg/L

Caption: CAS = Chemical Abstracts Service; pKa = logarithm of the acidity constant; Log KOW = logarithm of the n-octanol-water partition coefficient.

**Table 2:** Composition of the commercial ketoprofen-based formulations used in the study.

Identification	Composition
KET-1	Ketoprofen 100 mg; microcrystalline cellulose; Copovidone; Silicon dioxide; Magnesium stearate; Lactose monohydrate; Simethicone; Titanium dioxide; Quinoline yellow; Methacrylic acid copolymer; Talc; Triethyl citrate; Sodium bicarbonate, Sodium lauryl sulfate, Hypromellose and Macrogol.
KET-2	Ketoprofen 50 mg; Lactose monohydrate; Magnesium stearate and Croscarmellose sodium.
KET-3	Ketoprofen 150 mg; Dibasic calcium phosphate Dihydrate; Lactose monohydrate; Starch; Hydroxyethylcellulose; Iron oxide; Silicon dioxide and Magnesium stearate.
KET-4	Ketoprofen lysineate 320 mg; Carbomer 934; Magnesium stearate; Povidone; Talc; Eudragit and Diethyl phthalate.

**Table 3:** Treatments, pharmaceutical forms, ketoprofen concentrations, and test concentrations used in toxicity assays with *Microystis novacekii* and *Chlorella vulgaris*.

Treatment	Pharmaceutical Form	KET Concentration	Test Concentrations (mg/L)
API	Ketoprofen standard	≥98%	0.5; 5.0; 15.0; 25.0; 35.0; 45.0
KET-1	Commercial formulation 1	100 mg/tablet	0.5; 5.0; 15.0; 25.0; 35.0; 45.0
KET-2	Commercial formulation 2	50 mg/capsule	0.5; 5.0; 15.0; 25.0; 35.0; 45.0
KET-3	Commercial formulation 3	150 mg/tablet	0.5; 5.0; 15.0; 25.0; 35.0; 45.0
KET-4	Commercial formulation 4	320 mg/capsule	0.5; 5.0; 15.0; 25.0; 35.0; 45.0

**Table 4:** Risk classification derived from EC50 values obtained in ecotoxicological assays with cyanobacteria, following the criteria defined in Annex 2.28(B) of the Globally Harmonized System for the Classification and Labelling of Chemicals (GHS).

EC50 Value (X)	Toxicity Classification
X ≤ 1.0 mg/L	Highly toxic
1.0 < X ≤ 10.0 mg/L	Toxic
10.0 < X ≤ 100.0 mg/L	Low toxicity
X > 100 mg/L	Virtually non-toxic

**Table 5:** Results of the EC50 values from the toxicological evaluation of the active pharmaceutical ingredient (API) and the commercial ketoprofen-based drug (KET) on the cyanobacterium *Microcystis novacekii*.

ID	Producer	API	Exhibition	EC50 (mg/L)	Risk Classification	Statistical Model
API KET	Sigma	KET	4 days	3.22 ± 0.21	Toxic	log-logistic
API KET	Sigma	KET	14 days	1.35 ± 0.15	Toxic	log-logistic
KET-1	Medley	KET	4 days	C_MAX_ (45.0)	NA	NA
KET-1	Medley	KET	14 days	5.48 ± 1.41	Toxic	Weibull
KET-2	EMS	KET	4 days	C_MAX_ (45.0)	NA	NA
KET-2	EMS	KET	14 days	15.27 ± 1.07	Low toxicity	log-logistic
KET-3	Momenta	KET	4 days	32.44 ± 5.12	Low toxicity	Weibull
KET-3	Momenta	KET	14 days	5.85 ± 1.33	Toxic	Weibull
KET-4	Aché	LKET	4 days	31.28 ± 3.44	Low toxicity	log-logistic
KET-4	Aché	LKET	14 days	11.19 ± 0.82	Low toxicity	Weibull

Caption: EC50 = Concentration of effect (inhibition of cell growth) on 50% of organisms; C_MAX_ = maximum test concentration; KET = Ketoprofen; LKET = ketoprofen lysineate; NA = not applicable; ID = identification of the test substance; API = active pharmaceutical ingredient.

**Table 6:** Results of the EC50 values from the toxicological evaluation of the active pharmaceutical ingredient (API) and the commercial ketoprofen-based drug (KET) on the microalga *Chlorella vulgaris*.

ID	Producer	API	Exhibition	EC50 (mg/L)	Risk Classification	Statistical Model
API KET	Sigma	KET	4 days	5.05 ± 0.66	Toxic	Weibull
API KET	Sigma	KET	14 days	15.66 ± 1.34	Low toxicity	Weibull
KET-1	Medley	KET	4 days	C_MAX_ (45.0)	NA	NA
KET-1	Medley	KET	14 days	32.10 ± 3.22	Low toxicity	log-logistic
KET-2	EMS	KET	4 days	C_MAX_ (45.0)	NA	NA
KET-2	EMS	KET	14 days	30.01 ± 2.45	Low toxicity	log-logistic
KET-3	Momenta	KET	4 days	C_MAX_ (45.0)	NA	NA
KET-3	Momenta	KET	14 days	38.32 ± 2.00	Low toxicity	log-logistic
KET-4	Aché	LKET	4 days	C_MAX_ (45.0)	NA	NA
KET-4	Aché	LKET	14 days	C_MAX_ (45.0)	NA	NA

Caption: EC50 = Concentration of effect (inhibition of cell growth) on 50% of organisms; C_MAX_ = maximum test concentration; KET = Ketoprofen; LKET = ketoprofen lysineate; NA = not applicable; ID = identification of the test substance; API = active pharmaceutical ingredient.

**Table 7:** Risk Quotients (RQs) for ketoprofen in different water matrices based on Predicted No-Effect Concentrations (PNECs) derived from *Microcystis novacekii* (1.35 µg/L) and *Chlorella vulgaris* (15.66 µg/L), using global monitoring data [32].

Water Type	*n*	Concentration (µg/L) Mean (Min and Max)	RQ-1	RQ-2	Risk Classification
Drinkable	15	0.131 (0.001–0.561)	0.42	0.04	Moderate-Low
Superficial	566	0.267 (0.001–83.33)	>1.0	>1.0	High-High
Underground	42	0.049 (0.002–0.732)	0.54	0.05	Moderate-Low
Hospital effluent	4	0.323 (0.010–1.500)	>1.0	0.10	High-Moderate
Hospital influent	43	338.512 (0.032–14,200.00)	>1.0	>1.0	High-High
WWTP effluent	224	1.198 (0.001–270.76)	>1.0	>1.0	High-High
WWTP influent	398	18.015 (0.001–2747.29)	>1.0	>1.0	High-High

Caption: WWTP = wastewater treatment plant; RQ-1 = Risk Quotients *M. novacekii*; RQ-2 = Risk Quotients *C. vulgaris*; For the RQ calculation, the max value from the MEC was used: *n* = number of quantified samples.

## Data Availability

The data that support the findings of this study are available from the corresponding author upon reasonable request.

## Abbreviations

The following abbreviations are used in this manuscript:

KET	Ketoprofen
API	Active Pharmaceutical Ingredient
RQ	Risk quotient
NSAIDs	Nonsteroidal anti-inflammatory drugs
COX	Cyclooxygenase
OECD	Organisation for Economic Co-operation and Development
GHS	Globally Harmonised System
EC50	Half Maximal Effective Concentration
PNEC	Predicted Non-Effect Concentration
MEC	Measured Environmental Concentration
ANOVA	Analysis of Variance
NOEC	No Observed Effect Concentration
LOEC	Lowest Observed Effect Concentration
ROS	Reactive Oxygen Species
WWTP	Wastewater Treatment Plant Influents
